# Integrated Profiles of Transcriptome and mRNA m6A Modification Reveal the Intestinal Cytotoxicity of Aflatoxin B1 on HCT116 Cells

**DOI:** 10.3390/genes14010079

**Published:** 2022-12-27

**Authors:** Yajiao Wu, Wenqiang Bao, Jinjin Ren, Chutao Li, Mengting Chen, Dongcheng Zhang, An Zhu

**Affiliations:** 1 Key Laboratory of Ministry of Education for Gastrointestinal Cancer, School of Basic Medical Sciences, Fujian Medical University, Fuzhou 350108, China; 2 Department of Pathogen Biology, School of Basic Medical Sciences, Fujian Medical University, Fuzhou 350108, China; 3 Department of Biochemistry and Molecular Biology, School of Basic Medical Sciences, Fujian Medical University, Fuzhou 350108, China

**Keywords:** AFB1, m6A modification, transcriptome, endoplasmic reticulum stress, cell cycle, mitophagy, tight junction

## Abstract

Aflatoxin B1 (AFB1) is widely prevalent in foods and animal feeds and is one of the most toxic and carcinogenic aflatoxin subtypes. Existing studies have proved that the intestine is targeted by AFB1, and adverse organic effects have been observed. This study aimed to investigate the relationship between AFB1-induced intestinal toxicity and N6-methyladenosine (m6A) RNA methylation, which involves the post-transcriptional regulation of mRNA expression. The transcriptome-wide m6A methylome and transcriptome profiles in human intestinal cells treated with AFB1 are presented. Methylated RNA immunoprecipitation sequencing and mRNA sequencing were carried out to determine the distinctions in m6A methylation and different genes expressed in AFB1-induced intestinal toxicity. The results showed that there were 2289 overlapping genes of the differentially expressed mRNAs and differentially m6A-methylation-modified mRNAs. After enrichment of the signaling pathways and biological processes, these genes participated in the terms of the cell cycle, endoplasmic reticulum, tight junction, and mitophagy. In conclusion, the study demonstrated that AFB1-induced HCT116 injury was related to the disruptions to the levels of m6A methylation modifications of target genes and the abnormal expression of m6A regulators.

## 1. Introduction

Aflatoxins (AFTs) are natural toxins produced by fungi such as *Aspergillus flavus* and *Aspergillus parasiticus* and they are widely present in the production and preservation of tree nuts, cereal crops, spices, and animal feed [1]. The concentration of AFTs in cereal products intended for human consumption should not exceed 20 μg/kg according to U.S. food supervision system, while the maximum limited dosage was 4 μg/kg for cereals foods in the European Union [2]. According to global mycotoxin occurrence data, in the 74,821 feed samples collected from 100 countries, 23% of them were positive for AFTs, with the positive samples showing a median concentration of 4 µg/kg. Among the regions where aflatoxin B1 (AFB1) concentrations exceed 20 µg/kg, south Asia, sub-Saharan Africa, and southeast Asia have reported the highest positivity ratios of 41.1%, 38.5%, and 20.9%, respectively [3]. The worldwide AFB1 occurrence in highly used food commodities during 2008 to 2018 was reported, and sorghum, spices, and rice had the highest frequencies of 67.3%, 64.4%, and 54.5%, respectively; among the positive samples, the concentration ranges were 0.2–83.6, 0.2–25.4, and 0.76–73.2 µg/kg, respectively [4]. AFB1 can be transferred to offspring during pregnancy or lactation or spread in foods such as milk, eggs, and meat, thus indirectly endangering the health of humans and animals [1]. AFB1 remains a considerable socioeconomic and health problem worldwide.

More than 20 subtypes of AFTs have been isolated, including B1, B2, G1, G2, M1, and M2. These are structurally similar compounds composed of a dihydrofuran ring and a coumarin. Among these subtypes, AFB1 is the most harmful and carcinogenic. It has been proven that AFB1 is carcinogenic to humans, so it is categorized as the first-grade carcinogen [5]. The carcinogenicity of AFB1 mainly results from the activation of cytochrome P450 in the liver, which is the main toxicological mechanism in the liver [6]. The understanding of the toxicity of AFB1 is gradually deepening, and target organs besides the liver have been reported. Harrison et al. reported that colorectal tumor tissue had higher levels of adducts than normal tissue from the same patients with histories of AFB1 exposure [7]. However, how AFB1 exerts toxicity in the intestine remains unknown, and few studies have been devoted to elucidating this.

It has been proven that aflatoxin-induced toxicity is associated with m6A methylation modifications. AFB1 could trigger the reactive oxygen species (ROS) accumulation, followed by an increased abundance of m6A modifications, and result in hepatotoxicity in mice [8]. So far, various RNA modification types have been reported [9], including N1-methyladenosine [10], 5-methylcytosine [11], N6-methyladenosine (m6A) [12], and 7-methyl guanosine [13]. Among them, m6A is considered to be the most pervasive and abundant modification in eukaryotes. About 25% of mRNA has at least one m6A modification site [14]. The m6A modification regulates the translation, stability, transportation, and decay of mRNA in the post-transcriptional level, thus playing a critical role in biological processes and functions, such as the DNA damage response, embryo development, and primary miRNA processing [15,16]. In general, RNA m6A modifications are regulated by three enzymes [17]. Initially, RNA m6A modifications are mediated by methyltransferases. However, methylation can be removed by demethylases. The high-throughput methylated RNA immunoprecipitation sequencing (MeRIP-seq) technology has deepened the investigation of RNA modifications [18]. The m6A modifications typically occur in specific sites of RNA and have a preferred base sequence.

In the present study, the HCT116 cell line was selected as the intestinal experimental model, and MeRIP-seq was performed to provide a genome-wide overview of the toxicological responses of HCT116 cells treated with AFB1. The underlying toxicological mechanisms were explored based on bioinformatic analysis.

## 2. Materials and Methods

### 2.1. Chemical Reagent and Cell Culture

**Figure 1:**
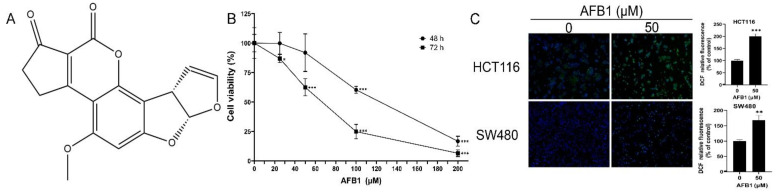
(**A**) The chemical structure of AFB1; (**B**) cell viability of HCT116 cells treated with AFB1; (**C**) representative fluorescence images of ROS accumulation and quantification analysis of fluorescence intensity. * p < 0.05, ** p < 0.01 or *** p < 0.001 indicates statistically significant difference.

The chemical structure of AFB1 (C_17_H_12_O_6_) was identified by nuclear magnetic resonance (Figure 1A). The purity of the AFB1 was 98.275%. The human intestinal cell lines HCT116 and SW480 were grown in DMEM (BasalMedia, Shanghai, China), containing 10% fetal bovine serum (Gibco, New York, NY, USA) and antibiotics. They were maintained with 5% CO_2_ at 37 °C. AFB1 in dimethyl sulfoxide (DMSO) was used for further experiments. The concentration of DMSO contained did not exceed 1%.

### 2.2. Cell Viability

The HCT116 cells were treated with 0, 25, 50, 100, and 200 μM AFB1 for 48 or 72 h. The cell viability was measured using the CCK-8 assay. In brief, after treatment of AFB1, the CCK-8 solution was added for incubation at 37 °C for 2 h and followed by the measurement of absorbance at 450 nm of a microplate reader (BioTek, Santa Clara, CA, USA).

### 2.3. RNA Preparation and m6A MeRIP-Seq of HCT116

After treatment with 50 μM AFB1 for 72 h, the HCT116 cells were lysed by TRIzol (Invitrogen, Carlsbad, CA, USA). The total RNA was extracted, and the concentration and integrity were quantified. The enrichment of RNAs with m6A methylation modifications and sequencing were performed by Seqhealth (Wuhan, China). Briefly, polyadenylated RNA enrichment was conducted by beads from 10 μg of total RNA. Then, mRNA was cleaved into fragments of 100–200 nt. The 0.5 mg/mL m6A antibody (Synaptic Systems, Goettingen, Germany) was used for m6A IP. Consequently, the RNA library was constructed for sequencing in DNBSEQ-T7 (MGI Technology Co., Ltd., Shenzhen, China).

### 2.4. Bioinformatic Analysis

The data were analyzed in Trim Galore and mapped to hg19 genome [19] with default parameters. The RNA expression level and the differential expression were analyzed by StringTie (Baltimore, MD, USA) [20] and DEseq [21], respectively. The m6A peak calling were identified using exomepeak2 [22,23]. STREME [24] was applied to identify the m6A motif sequences. The position weight matrix Markov model was integrated in the STREME algorithm. MetaTX [25] (Suzhou, China) was utilized to visualize the distribution of the epitranscriptome profiles. The m6A genes of the healthy human colon were obtained from m6A-TSHub [26]. The GO annotations and KEGG analysis were performed using the DAVID database (Frederick, MD, USA) [27]. The result of m6A conservation and disease association were obtained from ConsRM and RMDisease (Suzhou, China) [28,29]. The substrates of m6A regulators were obtained using starBase v2.0 (Guangzhou, China) [30].

### 2.5. Molecular Docking

Molecular docking was performed in SYBYL (Tripos, St Louis, MO, USA) to explore whether differentially expressed m6A regulators could interact with AFB1. The structures of human proteins were obtained from AlphaFold (DeepMind, London, UK) [31] and PDB and were preprocessed by applying SYBYL to extract ligand substructures, add hydrogen atoms, remove heteroatoms and water molecules, and carry out termini treatment [32]. The AFB1 structure was retrieved from PubChem, then converted to 3D structures using Chem3D (Waltham, MA, USA) and saved as a mol2 file. The AFB1 structure was set in an energy-minimized status [33]. The proteins were docked using the semi-flexible docking method in Surflex-Dock Geom mode. When the total score was higher than 5, the interaction between proteins and molecules was considered a stable model [34].

### 2.6. Intracellular ROS Accumulation

The intracellular ROS was evaluated after cells were treated with 50 μM AFB1 for 72 h. Briefly, the cells were incubated with DCFH-DA probe then washed with PBS to remove probes which did not enter the cells, followed by Hoechst 33342 for nuclear staining. The fluorescence images were analyzed in ImageJ to quantify the level of intracellular ROS.

### 2.7. Quantitative Real-Time PCR

The total RNA was extracted and reverse transcribed into cDNA. The AriaMx (Agilent, Palo Alto, CA, USA) provided quantitative detection and data analysis of mRNA expression levels. The human IGF2BP3 primers sequences used in RT-qPCR were as follows (5′-3′): forward GAGGCGCTTTCAGGTAAAATAG, reverse AATGAGGCGGGATATTTCGTAT.

### 2.8. Western Blotting

Cells were lysed with RIPA and the protein concentrations were measured by bicinchoninic acid. Then the proteins were separated and transferred onto the membranes. After blocking with skimmed milk, the membranes were incubated with primary and secondary antibodies (Proteintech and Abclonal, Wuhan, China), and then exposed with ECL reagents (Advansta, San Jose, CA, USA).

### 2.9. Statistical Analysis

The data analysis was conducted by SPSS (IBM, New York, NY, USA). One-way analysis of variance was performed. Difference of *p* < 0.05 was considered statistically significant. At least three independent experiments were performed in each assay.

## 3. Results

### 3.1. Cell Viability and ROS Accumulation

The cytotoxic effects of AFB1 on HCT116 cells were measured. As shown in Figure 1B, the AFB1-induced inhibition of cell viability was shown to be dose- and time-dependent. At 72 h, the cell viability was 62.4% in the 50 μM AFB1 group. The effect of ROS accumulation on HCT116 and SW480 cells induced by AFB1 was measured by using a DCFH-DA fluorescent probe. ROS accumulations were significantly increased in the AFB1 groups (Figure 1C).

### 3.2. Transcriptome-Wide MeRIP-Seq Reveals m6A Modification Pattern after AFB1 Treatment of HCT116 Cells

**Figure 2:**
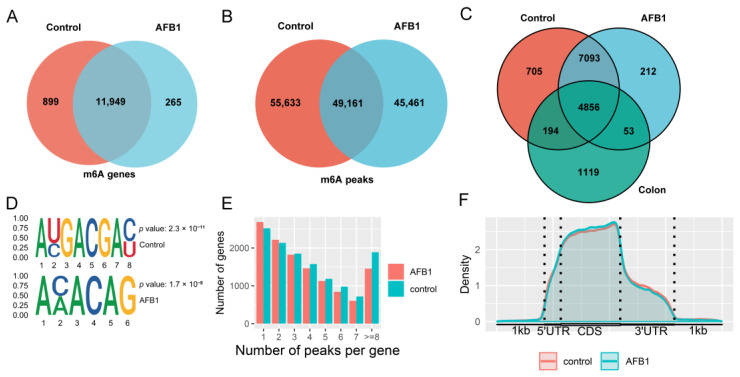
Venn diagram showing the (**A**) m6A genes and (**B**) peaks; (**C**) Venn diagram showing the m6A genes from the HCT116 control group, AFB1 group, and the healthy human colon data obtained by m6A-TSHub; (**D**) the motifs enriched from identified m6A peaks based on STREME; (**E**) the m6A-modified peaks number in genes; (**F**) the density of m6A-modified peaks in mRNA transcripts.

The summary of reads in MeRIP-seq were shown in Table S1. A total of 104,794 peaks were found in 12,848 genes of the control group, and 94,622 m6A peaks were found in 12,214 m6A genes of the AFB1 group. In the analysis of m6A-TSHub, 6222 m6A genes were reported in the healthy human colon (Figure 2A–C). The STREME was used to define the conserved sequence of RRACH, and the results in both the control and AFB1 groups showed the classical motifs (Figure 2D). To identify the distribution of m6A modifications throughout the transcriptome, the m6A-methylated transcripts were quantified in each gene, and it was found that more than 4000 genes contained 1–2 m6A peaks in each group (Figure 2E).

### 3.3. Analysis of m6A Modification Distribution

The distribution pattern of m6A methylation was analyzed in the control and AFB1 groups. As a result, the m6A peaks density were increased between 5′ untranslated regions (5′-UTRs) and the start codon region and remained high in the coding sequences (CDS) region (Figure 2F).

### 3.4. Differentially Expressed Genes (DEGs) and Differentially Methylated Genes

**Figure 3:**
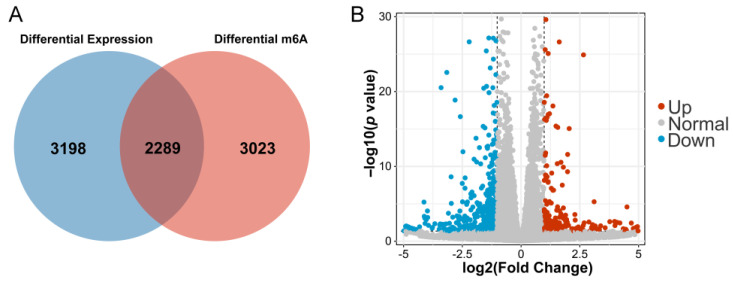
(**A**) DEGs and differentially m6A-modified mRNAs between cells with or without AFB1 treatment; (**B**) the volcano plot of 680 DEGs.

A total of 5487 DEGs were identified through the RNA-seq data and 5312 differentially m6A-modified mRNAs were identified between the control and AFB1 groups after the analysis of the MeRIP-seq data (*p* < 0.05). A total of 2289 genes were repeatedly detected as overlapping (Figure 3A). When the statistical threshold was adjusted to fold change > 2 and *p* < 0.05, a total of 248 mRNAs were up-regulated and 432 mRNAs were down-regulated (Figure 3B).

### 3.5. Biological Pathways of DEGs and Differentially m6A-Modified mRNAs

**Figure 4:**
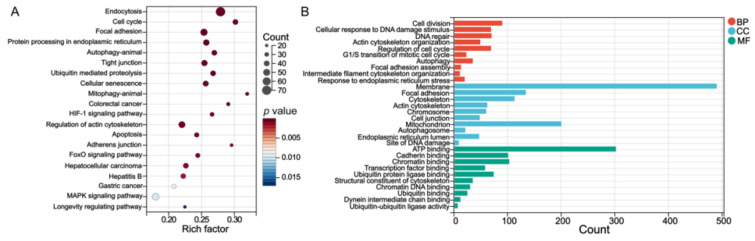
(**A**) The KEGG enrichment pathways and (**B**) GO terms of 2289 overlapped genes of DEGs and differentially m6A-methylated mRNAs.

KEGG and GO enrichment was performed using the DAVID bioinformatic database. The GO enrichment analysis contained subtypes of biological process (BP), cellular component (CC), and molecular function (MF). KEGG of 2289 overlapping genes was involved in the cell cycle, endoplasmic reticulum (ER), autophagy, tight junction, mitophagy, and apoptosis (Figure 4A). The BP terms were cell cycle, autophagy, and response to ER stress. The CC terms included focal adhesion, cell junction, mitochondrion, and ER lumen. The MF terms contained cadherin binding (Figure 4B).

### 3.6. Conservation and Disease Association of m6A-Modified Genes

**Figure 5:**
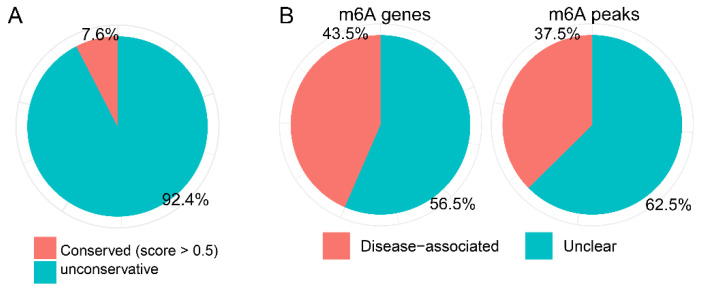
The (**A**) conservation of m6A sites and (**B**) disease association of the 2289 overlapping genes.

The conservation of m6A sites and disease association were explored in the 2289 overlapping genes. The result showed that 92.4% of the m6A-methylation-modified sites were non-conserved, and 43.5% genes and 37.5% peaks were diseased-associated (Figure 5).

### 3.7. Genes Expression and Their Potential m6A Regulators

After the AFB1 treatment in HCT116 cells, the mRNA expression levels related to cell cycle (*MYC*, *CCNB1*, *CDC25C*, *ATM*, and *CHEK2*), protein processing in ER (*PDIA3*, *HSPA5*, *P4HB*, *HSP90B1*, and *CANX*), tight junction (*MYH9*, *ACTB*, *ACTG1*, *ACTR2*, and *SRC*), and mitophagy (*BECN1*, *MFN2*, *GABARAPL1*, *MAP1LC3B*, and *SQSTM1*) were abnormal. (Table 1). The m6A regulators were identified using the CLIP technique, including several writers and readers.

### 3.8. Proteins Expression Levels

**Figure 6:**
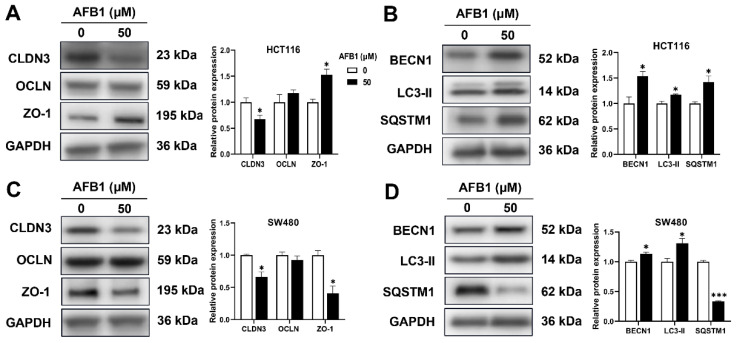
The proteins expression of CLDN3, OCLN, ZO-1, BECN1, LC3-II, and SQSTM1 in AFB1-treated HCT116 (**A**,**B**) and SW480 cells (**C**,**D**). * p < 0.05 or *** p < 0.001 indicates statistically significant difference.

In the Western blotting assay, tight junction and mitophagy biomarkers were detected. As shown in Figure 6, after treatment of AFB1, the CLDN3 was decreased in HCT116 cells, and ZO-1, BECN1, LC3-II and SQSTM1 were increased. The CLDN3, ZO-1 and SQSTM1 were decreased, and BECN1 and LC3-II were increased in SW480 cells. The OCLN was not altered by AFB1 in HCT116 and SW480 cells.

### 3.9. Potential RNA m6A Regulators of Differentially Methylated Genes

**Figure 7:**
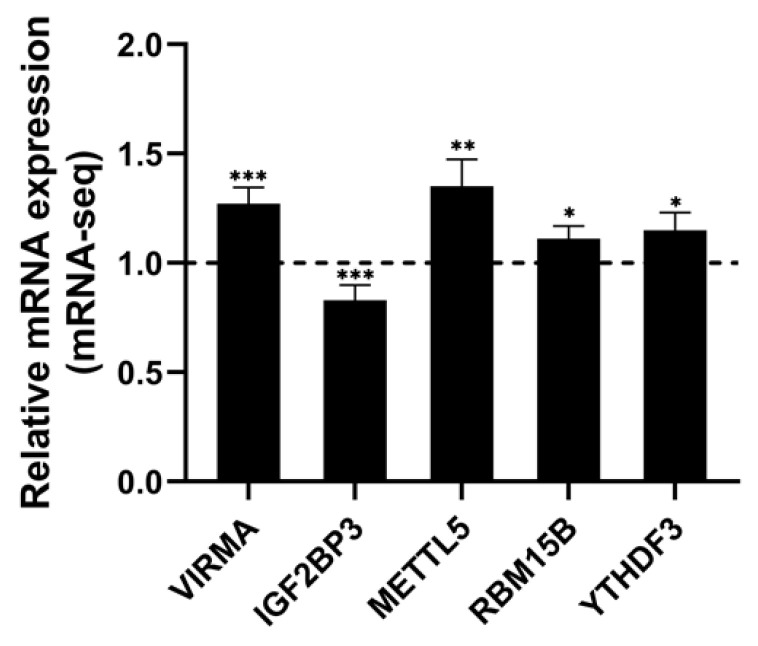
The significantly differential expressed m6A regulators in HCT116 with or without AFB1 treatment. Data were expressed as means ± SD. * *p* < 0.05, ** *p* < 0.01, *** *p* < 0.001 or *p* < 0.0001 versus the control.

Through the analysis of mRNA-seq data, a total of 23 mRNA of m6A regulators were differentially expressed (Table S2 and Figure 7). The writers *VIRMA, METTL5*, *RBM15B*, and *ZC3H13* were upregulated, and the reader *IGF2BP3* was downregulated (Figure 7). The mRNA expression of IGF2BP3 has been verified by RT-qPCR in AFB1-treated HCT116 and SW480 cells (Figure S1).

**Figure 8:**
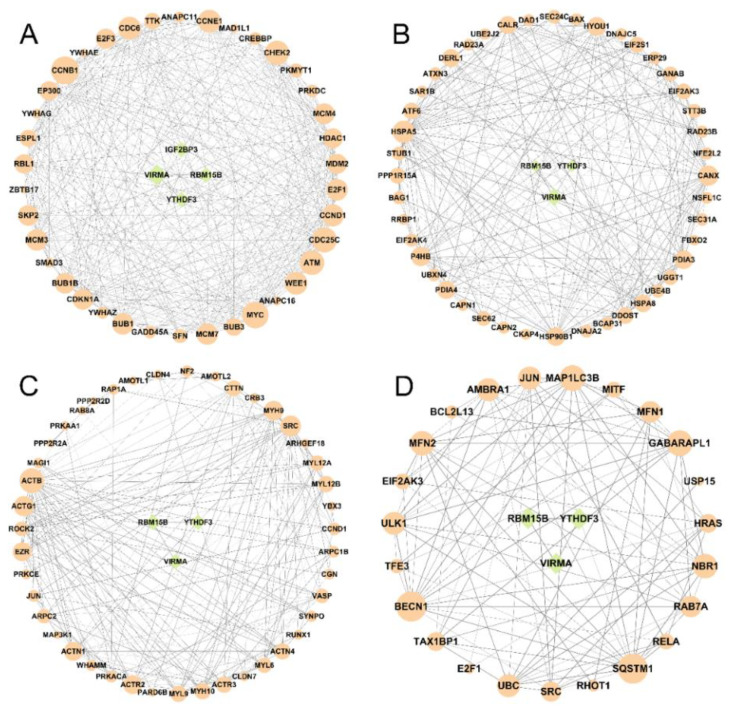
The interactions between m6A regulators (green circle) and the genes (orange circle) from the KEGG enrichment pathways of (**A**) cell cycle, (**B**) ER, (**C**) tight junction, and (**D**) mitophagy.

The GO and KEGG terms were enriched in cell cycle, ER, tight junction, and mitophagy. We collected the genes that were enriched in the mentioned terms and explored the regulatory relationship between differentially expressed m6A regulators and these target genes by STRING to perform protein–protein interactions network analysis. As shown in Figure 8, the top five genes in cell cycle terms were *CCNB1*, *MYC*, *CDC25C*, *ATM*, and *CHEK2*, with the degrees of 33, 31, 29, 28, and 28 (Figure 8A); in terms of protein processing in ER, the top five genes were *HSPA5*, *CANX*, *P4HB*, *HSP90B1*, and *PDIA3*, with the degrees of 30, 25, 23, 21, and 21 (Figure 8B); in the tight junction terms, the top five genes were *ACTB*, *SRC*, *ACTG1*, *MYH9*, and *ACTR23*, with the degrees of 27, 21, 20, 19, and 18 (Figure 8C); in the mitophagy terms, the top five genes were *BECN1*, *SQSTM1*, *GABARAPL1*, *MAP1LC3B*, and *MFN2*, with the degrees of 16, 16, 13, 13, and 12 (Figure 8D). The gene set enrichment analysis of these four terms in HCT116 cells treated with AFB1 were shown in Figure S2.

**Figure 9:**
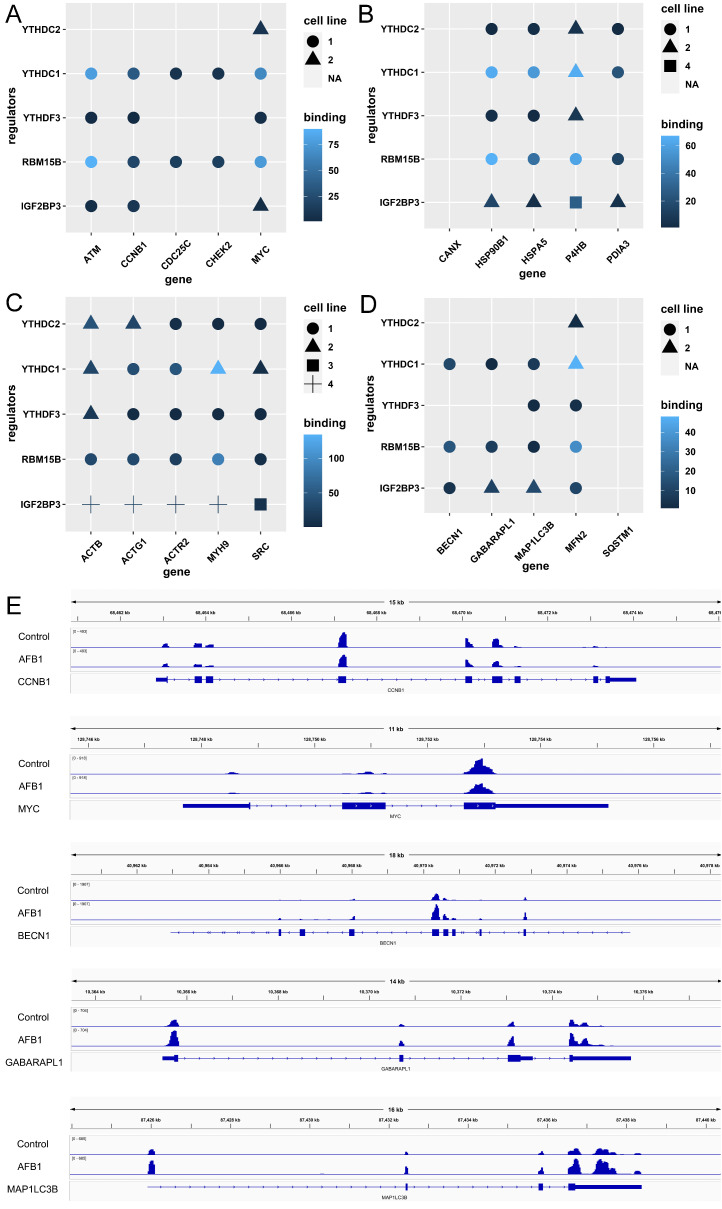
The substrates of m6A regulators: (**A**) cell cycle, (**B**) ER, (**C**) tight junction, and (**D**) mitophagy pathways enriched in the KEGG analysis. The cell lines including HeLa, Huh7, HEK293, HepG2, Panc1, PL45, etc. were from the starBase v2.0. (**E**) depicts the levels of m6A methylation on *CCNB1*, *MYC*, *BECN1*, *GABARAPL1*, and *MAP1LC3B* mRNA transcripts.

To detect the substrates of m6A methylation regulators, the writers RBM15B, VIRMA, METTL5, and ZC3H13 and the readers YTHDC2, YTHDC1, YTHDF3, and IGF2BP3 were selected as well as the cell cycle genes *CCNB1*, *MYC*, *CDC25C*, *ATM*, and *CHEK2* (Figure 9A); the genes of protein processing in ER *HSPA5*, *CANX*, *P4HB*, *HSP90B1*, and *PDIA3* (Figure 9B); the tight junction genes A*CTB*, *SRC*, *ACTG1*, *MYH9*, and *ACTR2* (Figure 9C); and the mitophagy genes *BECN1*, *SQSTM1*, *GABARAPL1*, *MAP1LC3B*, and *MFN2* (Figure 9D). The m6A methylation levels of transcripts were observed by IGV and shown in Figure 9E.

### 3.10. Interactions between AFB1 and m6A Regulators

**Figure 10:**
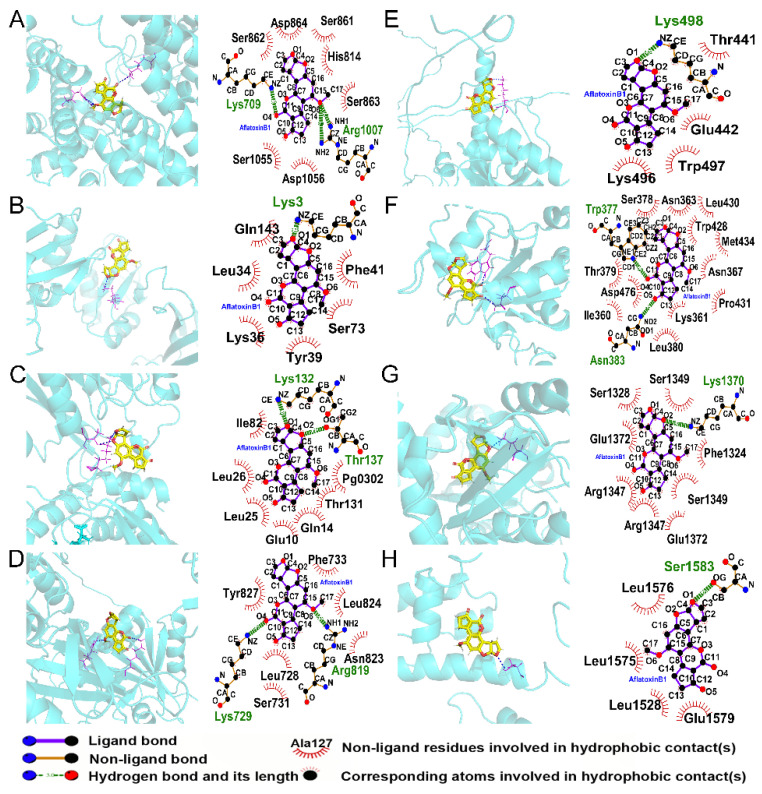
The interactions between AFB1 and m6A regulators of (**A**) VIRMA, (**B**) IGF2BP3, (**C**) METTL5, (**D**) RBM15B, (**E**) YTHDF3, (**F**) YTHDC1, (**G**) YTHDC2, and (**H**) ZC3H13.

The interactions between AFB1 and m6A regulators were predicted and shown in Table 2 and Figure 10. Theoretically, AFB1 bound to regulators by hydrogen bonds and hydrophobic contacts. All the total scores except ZC3H13 were higher than 5, indicating that most of these proteins could form stable and direct interactions with AFB1.

## 4. Discussion

Generally, the digestive system represents the first barrier against exposure to foods contaminated with AFB1, so the biological interaction between the intestine and AFB1 is an important issue to be clearly elucidated. To date, most studies related to the toxicity of AFB1 have focused on the metabolic process and hepatotoxicity. The liver and hepatocytes are the main experimental models in toxicological research. However, the liver is not the sole toxicity target of AFB1, and the intestine injury induced by AFB1 merits more attention. In this study, human intestinal cells were exposed to AFB1, and the alterations of m6A modifications and the mRNA expression levels were analyzed through MeRIP-seq.

According to the present sequencing data, the m6A-modified transcripts were mainly enriched near the stop codons, which were consistent with the topology of human RNA m6A methylomes [35]. Additionally, by exploring the distribution of m6A-modified peaks, it was found that most genes contain one or two m6A peaks, which was consistent with a previous description [15].

The KEGG enrichments reported terms of cell cycle, focal adhesion, protein processing in ER, autophagy, mitophagy, hepatitis B, etc. Many vital terms were enriched based on GO analysis. The cell cycle, autophagy, and ER stress were enriched in BP; the actin cytoskeleton, mitochondrion, autophagosome, and ER lumen were enriched in CC; and ATP binding, cadherin binding, and ubiquitin protein ligase binding were enriched in MF. The intestine is particularly susceptible to xenobiotic compounds. When the intestine is exposed to mycotoxins, the intestinal epithelial barrier, which consists of various cellular junctions, is regarded as a mechanical barrier to the toxins [36]. The tight junction, focal adhesion, and adherens junction are important components of cellular junctions. The mycotoxins can affect tight junction proteins, thus compromising the integrity of the intestinal barrier [37]. In this study, the cellular junctions and actin cytoskeleton were damaged in HCT116 cells, which may have resulted in abnormal intestinal permeability, absorption, and efflux.

The toxicity of AFB1 is involved in ROS production, which is one of the main factors in the toxicity toward organs [38]. It has been proven that AFB1-induced oxidative stress can activate ER stress (ERS) [39]. The ER is the major membrane-bound organelle for intracellular calcium storage and protein synthesis, folding, and transport. ROS can initiate ERS and mediate the unfolded protein response, resulting in calcium imbalance [40]. Existing studies have reported that ERS could block the cell cycle to prevent daughter cell generation [41]. Previous studies also demonstrated that exposure to AFB1 could induce cell cycle arrest at different phases among diverse cells, such as arrest at the G0/G1 or S phases in human liver cells [42,43,44]. After the GO and KEGG enrichment in the present study, AFB1 was found to arrest cell cycle at G1/S transition, as shown in Figure 4B. In addition, the ER and mitochondria are highly interconnected organelles, since the ER membrane and the outer mitochondrial membrane are tightly connected to each other, and this region is called the mitochondria-associated ER membrane (MAM) [45]. ERS is associated with mitochondrial dysfunction. When the ER homeostasis is disrupted, calcium ions are released from the ER into the mitochondria, leading to the production of large amounts of ROS in the mitochondria, and mitochondrial bioenergetics are regulated by effecting the MAM. In turn, ER failure is accelerated, and mitochondrial damage is aggravated [46]. Then, autophagy is triggered to selectively identify and eliminate damaged mitochondria [47].

RNA m6A methylation involves in the post-transcriptional modification and affects diverse, fundamental biological processes and functions of mRNA, such as stability, translation, and decay. RNA m6A methylation is regulated by methyltransferases, demethylases, and methylation recognition proteins [48,49,50]. In this study, eight regulators were abnormally expressed. Among them, IGF2BP3 was the m6A-methylation-recognition protein that enhanced mRNA stability. As determined using computational prediction technology, AFB1 was able to bind stably to m6A regulators excluding ZC3H13. Then, this study explored the endpoint effects of the differentially expressed m6A regulator genes on the enriched pathways of cell cycle, mitophagy, and protein processing in the ER and tight junction. For example, in the term of cell cycle, cyclin B1 (CCNB1) and MYC promote the progression of the cell cycle [51,52]. For the term of mitophagy, BECN1 and GABA type A receptor-associated protein such as 1 (GABARAPL1) are involved in the formation of mature autophagosome membranes [53,54]. Once autophagy occurs, light chain 3 (LC3) acts as a biomarker, and the lipidation of LC3-I to LC3-II is observed during autophagosome formation [55]. For the term of protein processing in the ER, the heat shock proteins are essential molecular chaperones for regulating protein homeostasis both under normal physiological conditions and in ERS [56,57]. In this study, the m6A methylation levels of CCNB1 and MYC were reduced in the AFB1 group, along with decreased binding to IGF2BP3; conversely, the m6A methylation levels of BECN1, GABARAPL1, and MAP1LC3B were elevated, along with increased binding to IGF2BP3. As a result, the stability of CCNB1 and MYC mRNA was destroyed, leading to decreased mRNA expression and translation. On the contrary, the mRNAs of BECN1, GABARAPL1, and MAP1LC3B were highly expressed and translated.

## 5. Conclusions

In this study, the cytotoxicity of AFB1 toward HCT116 cells was observed. The potential intestinal toxicological effects of AFB1, and the GO and KEGG enriched terms of cell cycle, mitophagy, protein processing in ER, and tight junction, are reported. These injuries result in structural abnormalities and dysfunction of the intestine. This study suggests that the underlying molecular toxicological mechanisms are associated with mRNA m6A methylation; namely, AFB1 might decrease CCNB1 and MYC mRNA expression by reducing the levels of mRNA m6A methylation, resulting in cell cycle arrest. On the other hand, AFB1 induced mitophagy, probably by elevating the level of m6A methylation modifications, mRNA expression, and translation of BECN1, GABARAPL1, and MAP1LC3B. This study provides evidence of AFB1-induced intestinal cytotoxicity and a potential m6A epigenetic regulation mechanism for mRNA.

## Figures and Tables

**Table 1:** The mRNA expression and their potential substrates of m6A regulators.

	Gene	log2 Fold Change	*p* adj	Writer				Reader			
				VIRMA	METTL5	RBM15B	ZC3H13	IGF2BP3	YTHDF3	YTHDC1	YTHDC2
Cell cycle	*MYC*	−0.4058	4.49 × 10^−7^	N	N	Y	N	Y	Y	Y	Y
	*CCNB1*	−0.3427	5.27 × 10^−5^	N	N	Y	N	Y	Y	Y	N
	*CDC25C*	−0.6666	5.73 × 10^−4^	N	N	Y	N	N	N	Y	N
	*ATM*	−0.2175	4.34 × 10^−2^	N	N	Y	N	Y	Y	Y	N
	*CHEK2*	−0.2822	1.36 × 10^−1^	N	N	Y	N	N	N	Y	N
Protein processing in ER	*PDIA3*	−0.4593	1.74 × 10^−11^	N	N	Y	N	Y	N	Y	Y
	*HSPA5*	−0.3711	1.57 × 10^−7^	N	N	Y	N	Y	Y	Y	Y
	*P4HB*	−0.2804	2.17 × 10^−7^	N	N	Y	N	Y	Y	Y	Y
	*HSP90B1*	−0.2779	4.57 × 10^−3^	N	N	Y	N	Y	Y	Y	Y
	*CANX*	−0.2316	6.81 × 10^−2^	N	N	N	N	N	N	N	N
Tight junction	*MYH9*	0.5887	6.34 × 10^−15^	N	N	Y	N	Y	Y	Y	Y
	*ACTB*	0.3106	6.87 × 10^−9^	N	N	Y	N	Y	Y	Y	Y
	*ACTG1*	0.3115	4.92 × 10^−7^	N	N	Y	N	Y	Y	Y	Y
	*ACTR2*	0.2744	2.34 × 10^−2^	N	N	Y	N	Y	Y	Y	Y
	*SRC*	0.2087	6.32 × 10^−2^	N	N	Y	N	Y	Y	Y	Y
Mitophagy-animal	*BECN1*	0.4214	1.08 × 10^−8^	N	N	Y	N	Y	N	Y	N
	*MFN2*	0.2190	7.00 × 10^−4^	N	N	Y	N	Y	Y	Y	Y
	*GABARAPL1*	0.1765	9.41 × 10^−3^	N	N	Y	N	Y	N	Y	N
	*MAP1LC3B*	0.1570	3.93 × 10^−2^	N	N	Y	N	Y	Y	Y	N
	*SQSTM1*	0.1098	7.02 × 10^−2^	N	N	N	N	N	N	N	N

Y = Yes, and N = No. Whether the mRNA is the substrate of the m6A regulator.

**Table 2:** The predicted interactions between AFB1 and m6A regulators.

Protein	PDB ID	Total Score	Crash	Polar	H-Bond Number	Residues Involved in H-Bond Formation	Hydrophobic Contacts Number	Residues Involved in Hydrophobic Contacts
VIRMA	7VF5	6.8256	−0.4059	4.9085	3	Lys709, Arg1007 (2 Hydrogen bonds)	7	Ser862, Asp864, Ser861, His814, Ser863, Asp1056, Ser1055
IGF2BP3	6FQR	5.527	−0.7161	2.0492	1	Lys3	6	Phe41, Ser73, Tyr39, Lys36, Leu34, Gln143
METTL5	6H2V	5.6646	−1.2471	2.1114	2	Lys132, Thr137	7	Pg0302, Thr131, Gln14, Glu10, Leu25, Leu26, Ile82
RBM15B	Predicted by AlphaFold	5.2143	−1.1279	2.3631	2	Lys729, Arg819	6	Tyr827, Phe733, Leu824, Asn823, Leu728, Ser731
YTHDF3	Predicted by AlphaFold	5.3677	−0.9188	2.2975	1	Lys498	4	Thr441, Glu442, Trp497, Lys496
YTHDC1	6SZT	6.123	−1.5099	2.2817	2	Trp377, Asn383	12	Ser378, Asn363, Leu430, Trp428, Met434, Asn367, Pro431, Lys361, Leu380, Ile360, Asp476, Thr379
YTHDC2	6K6U	6.4879	−1.0525	1.4697	1	Lys1370	8	Ser1349, Ser1328, Glu1372, Arg1347, Arg1347, Glu1372, Ser1349, Phe1324
ZC3H13	7VF2	4.1247	−0.6071	0.8869	1	Ser1583	4	Leu1576, Leu1575, Leu1528, Glu1579

## Data Availability

All data are included in this manuscript.

## Supplementary Materials

The following supporting information can be downloaded at: https://www.mdpi.com/article/10.3390/genes14010079/s1, Figure S1: The mRNA expression levels of IGF2BP3 in AFB1-treated HCT116 and SW480 cells; Figure S2: The GSEA of (A) cell cycle, (B) protein processing in endoplasmic reticulum, (C) tight junction, and (D) mitophagy-animal in HCT116 cells treated with AFB1; Table S1: Summary of reads of MeRIP-seq and mRNA-seq in HCT116 cells with or without AFB1 treatment; Table S2: The mRNA expression levels of m6A regulators in AFB1-treated HCT116 cells.
