# Supplementary material for: Integrated Profiles of Transcriptome and mRNA m6A Modification Reveal the Intestinal Cytotoxicity of Aflatoxin B1 on HCT116 Cells
Source: Genes (Basel). 2022 Dec 27;14(1):79. doi: 10.3390/genes14010079 (PMC9858580; doi:10.3390/genes14010079)
Supplement: Supplementary file 1 [file genes-14-00079-s001.zip › genes-2009324-supplementary.pdf]

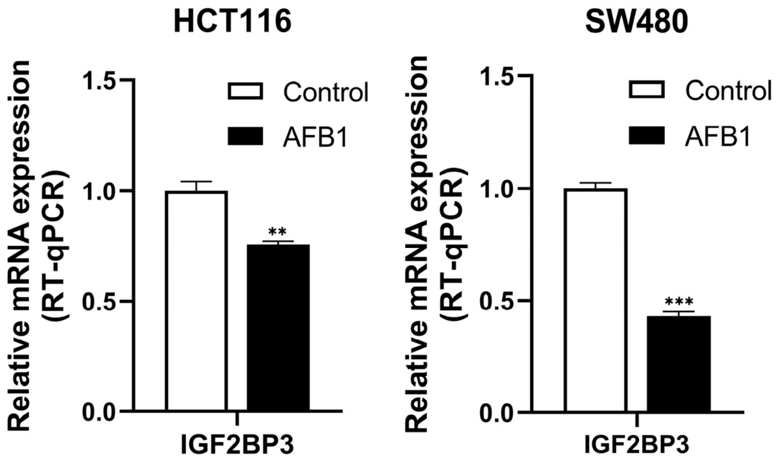

Figure S1. The mRNA expression levels of IGF2BP3 in AFB1-treated HCT116 and SW480 cells. Data were expressed as mean ± SEM, n=3. $^{**}p < 0.01$, $^{***}p < 0.0001$ versus the control.

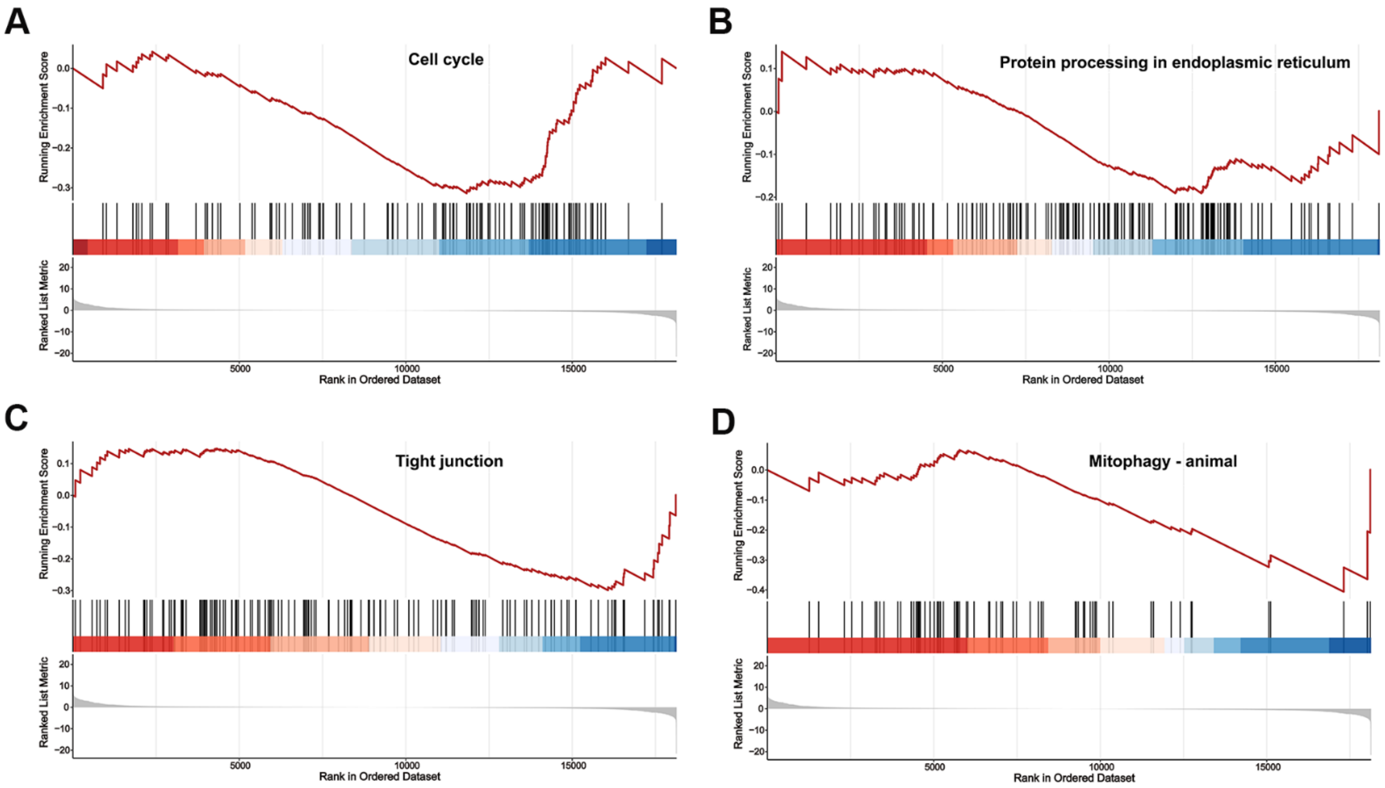

Figure S2. The GSEA of (**A**) cell cycle, (**B**) protein processing in endoplasmic reticulum, (**C**) tight junction, and (**D**) mitophagy-animal in HCT116 cells treated with AFB1.

Table S1. Summary of reads of MeRIP-seq and mRNA-seq in HCT116 cells with or without AFB1 treatment.

Sample	Raw reads	Raw bases (G)	Clean reads	Clean bases (G)	Effective rate (%)	Q20 (%)	Q30 (%)
Control_1_IP	45,955,030	6.89	37,356,030	4.61	81.29	99.07	96.39
Control_1_Input	57,354,896	8.60	47,078,964	5.89	82.08	99.13	96.65
Control_2_IP	72,201,732	10.83	59,867,556	7.38	82.92	99.09	96.46
Control_2_Input	57,813,808	8.67	46,979,252	6.14	81.26	99.04	96.31
Control_3_IP	79,366,256	11.90	67,414,202	8.22	84.94	99.22	96.92
Control_3_Input	64,346,344	9.65	54,475,288	6.87	84.66	99.18	96.80
AFB1_1_IP	66,338,140	9.95	56,452,100	7.03	85.10	99.17	96.75
AFB1_1_Input	48,935,274	7.34	32,976,916	4.06	67.39	99.10	96.55
AFB1_2_IP	72,110,258	10.82	62,770,108	7.99	87.05	99.34	97.31
AFB1_2_Input	61,696,986	9.25	49,932,164	6.15	80.93	99.04	96.35
AFB1_3_IP	71,026,356	10.65	58,153,984	7.23	81.88	99.08	96.45
AFB1_3_Input	64,254,764	9.64	51,796,188	6.48	80.61	99.00	96.25

Q20 and Q30 refers to the percentage of nucleotides with Phred quality score >20 and >30, respectively.

Table S2. The mRNA expression levels of m6A regulators in AFB1-treated HCT116 cells.

Gene	Regulation	Base Mean	log2 Fold Change	*p* adj	*p* value
YTHDC1	reader	8183.29	0.2698	5.01×10^{-2}	1.28×10^{-2}
YTHDC2	reader	1240.49	−0.2850	7.79×10^{-2}	2.25×10^{-2}
ZC3H13	writer	13,752.12	0.2560	1.07×10^{-1}	3.40×10^{-2}
FMR1	reader	2850.07	0.1846	1.84×10^{-1}	7.12×10^{-2}
HNRNPA2B1	reader	40,454.61	−0.1448	2.41×10^{-1}	1.03×10^{-1}
METTL14	writer	2843.99	0.1324	3.01×10^{-1}	1.41×10^{-1}
HNRNPC	reader	12,102.42	0.1168	3.10×10^{-1}	1.47×10^{-1}
FTO	eraser	1060.08	−0.1201	3.61×10^{-1}	1.83×10^{-1}
YTHDF2	reader	3660.90	0.0777	4.07×10^{-1}	2.18×10^{-1}
METTL16	writer	1133.56	−0.1061	4.64×10^{-1}	2.64×10^{-1}
IGF2BP2	reader	6558.11	−0.0900	5.66×10^{-1}	3.58×10^{-1}
ALKBH5	eraser	3169.13	−0.0906	5.91×10^{-1}	3.85×10^{-1}
RBM15	writer	1237.94	−0.0983	6.89×10^{-1}	4.96×10^{-1}
IGF2BP1	reader	19.57	−0.3703	7.07×10^{-1}	5.22×10^{-1}
METTL3	writer	1099.49	0.0409	7.86×10^{-1}	6.26×10^{-1}
CBLL1	writer	1211.29	0.0171	9.39×10^{-1}	8.79×10^{-1}
YTHDF1	reader	2315.30	0.0020	9.89×10^{-1}	9.77×10^{-1}
WTAP	writer	2420.62	−0.0016	9.93×10^{-1}	9.83×10^{-1}
